# Microencapsulation of Enteric Bacteriophages in a pH-Responsive Solid Oral Dosage Formulation Using a Scalable Membrane Emulsification Process

**DOI:** 10.3390/pharmaceutics11090475

**Published:** 2019-09-14

**Authors:** Gurinder K. Vinner, Kerry Richards, Miika Leppanen, Antonia P. Sagona, Danish J. Malik

**Affiliations:** 1 Chemical Engineering Department, Loughborough University, Loughborough LE11 3TU, UK; G.Vinner@lboro.ac.uk (G.K.V.); K.Richards@lboro.ac.uk (K.R.); 2 Department of Physics, University of Jyväskylä, Jyväskylä FI-40014, Finland; miika.j.leppanen@jyu.fi; 3 School of Life Sciences and Warwick Integrative Synthetic Biology Centre, University of Warwick, Coventry CV4 7AL, UK

**Keywords:** microencapsulation, bacteriophage therapy, controlled release, enteric infections, pH-triggered release, *E. coli*, Eudragit S100

## Abstract

A scalable low-shear membrane emulsification process was used to produce microencapsulated *Escherichia coli*-phages in a solid oral dosage form. Uniform pH-responsive composite microparticles (mean size ~100 µm) composed of Eudragit^®^ S100 and alginate were produced. The internal microstructure of the gelled microcapsules was studied using ion-milling and imaging, which showed that the microparticles had a solid internal core. The microencapsulation process significantly protected phages upon prolonged exposure to a simulated gastric acidic environment. Encapsulated phages that had been pre-exposed to simulated gastric acid were added to actively growing bacterial cells using in vitro cell cultures and were found to be effective in killing *E. coli*. Encapsulated phages were also shown to be effective in killing actively growing *E. coli* in the presence of human epithelial cells. Confocal microscopy images showed that the morphology of encapsulated phage-treated epithelial cells was considerably better than controls without phage treatment. The encapsulated phages were stable during refrigerated storage over a four-week period. The process of membrane emulsification is highly scalable and is a promising route to produce industrial quantities of pH-responsive oral solid dosage forms suitable for delivering high titres of viable phages to the gastrointestinal tract.

## 1. Introduction

The emergence of antibiotic resistance in bacteria is a serious global threat to human health. Common enteric bacterial pathogens have become progressively more resistant to conventional frontline antibiotics [1,2]. Treatment of antibiotic resistant bacterial infections is an urgent priority. National State Health Departments around the world are banning general antibiotic use in animal husbandry; alternative, safe, and low-cost biocontrol strategies to reduce pathogen carriage in livestock and poultry are urgently needed. Development of new classes of novel antibiotics are not keeping pace with the rate of antibiotic resistance [3]. Exploration of alternative treatment options are urgently needed [4]. Truly virulent bacteriophages (phages) are viruses that infect and kill bacteria in a highly specific manner. They represent a promising approach to targeting bacterial infections in a treatment known as phage therapy [5,6,7,8]. The specific interaction between bacteriophages and their bacterial hosts makes them an attractive alternative to employing broad spectrum antibiotics in modulating and maintaining a healthy gut microbiome [4,9]. In instances of enteric bacterial infections where the causative agent and strain may be suitably diagnosed, treatment with a sufficiently high initial phage dose could promote rapid in situ phage multiplication and killing of the targeted bacteria [10,11,12]. Enteric infections worldwide are typically caused by pathogens such as *Escherichia coli*, *Salmonella spp*, *Vibrio cholerae*, and *Clostridium difficile* [13]. Enteric pathogens could be promising candidates for the development of phage therapy; however, there are significant barriers to be overcome in terms of the logistics of delivering a stable, defined phage dose to the infection site [14].

There is likely to be a significant loss in the titre of orally administered phages by the time they reach the intended infection site [15]. Liquid phage formulations taken orally exposes phages to stomach acidity and digestive tract contents (enzymes such as pepsin and pancreatin), increasing the risk of phage viability loss [16]. A recent in vivo study in chickens showed a significant reduction (3 log reductions compared to the dose given) in viable phages reaching the gastrointestinal tract due to stomach acid exposure [17]. Mice receiving an oral dose (T4 coliphages in drinking water) of 10^9^ PFU/g gut contents had a thousand-fold lower phage titer, indicating a sizable loss in phage activity [16]. Phage inactivation attributed to stomach acidity may in part have been responsible for failure of a recent clinical trial in children using phage therapy to treat acute bacterial diarrhoea symptoms [14]. Acidity in the stomach as well as bile and digestive enzymes and other proteases in the intestinal tract and stomach are important environmental stresses for phage inactivation [18,19,20]. Oral application of phages in Georgia and Poland are typically preceded by gastric neutralization [21]. There is a clear need to protect phages against adverse gastrointestinal environmental conditions and to control their targeted release at the site of infection such as in the lower gastrointestinal tract (GIT) compartments of the cecum and colon for *E. coli* infections; this is achievable through encapsulation [19,20,22]. Microencapsulated phages may also result in longer transit times through the GIT in comparison with application of free phages due to mucoadhesive interactions with the gastrointestinal mucus [23] or in animals such as chickens, where retention of microparticles in the crop may result in slowing their transit through the GIT [17]. The purpose of encapsulation is to protect phages from harsh environmental stresses found in the gastrointestinal tract as well as to protect the phages during processing and storage prior to use [24], whilst yielding a product that is easy to handle and apply, e.g., with animal feed [17].

We report here for the first time the use of a scalable membrane emulsification platform technology to encapsulate phages suitable for targeted delivery to the gastrointestinal tract. Small microparticles with encapsulated *E. coli*-phage K1F in a composite ES100^®^ and alginate matrix were produced using microsieve membranes, which are arrays of uniform micropores. A novel aspect of the research presented here is demonstration of the suitability of microencapsulation of phages using a low-shear membrane emulsification process that allows the manufacture of uniform micron-sized, pH-responsive solid oral dosage forms using acidified oil for the precipitation of Eudragit^®^ S100 (ES100) and calcium ions to induce alginate gelation in solution. The technique affords control over the choice of formulation as well as morphology of the microparticles, including their size and size distribution; these influence phage release kinetics, phage loading, and encapsulation efficiency. The resulting pH-sensitive microcapsules were designed to survive the transition of phages delivered orally via the mouth and through the stomach, followed by phage release in the intestine. The small size of the microcapsules is particularly useful for efficacy testing of encapsulated phages via oral delivery using small-bore gavage tubes for testing in animal models such as in mice and rats.

## 2. Materials and Methods

### 2.1. Chemical Reagents

A methyl methacrylate co-methacrylic acid copolymer Eudragit^®^ S100 was bought from Evonik, Germany. Alginate (medium viscosity) was bought from Sigma Aldrich, Gillingham, UK. Miglyol 840 (propylene glycol diester of caprylic/capric acid) was bought from Safic-Alcan UK Ltd., Warrington, UK. Food grade Castor oil was purchased from Elf Foods, Loughborough, UK. Polyglycerol polyricinoleate (PGPR), which is an oil-soluble surfactant, was bought from Aston Chemicals Ltd., Aylesbury, UK. Calcium chloride, *p*-toluenesulfonic acid, Tween 20, and sodium chloride were bought from Fisher Scientific, Loughborough, UK. Sorensen’s buffer (0.2 M) was used to dissolve the microparticles. It was prepared by mixing different proportions of potassium phosphate monobasic (KH_2_PO_4_) with sodium phosphate dibasic (Na_2_HPO_4_) (for pH 7, 50 mL of KH_2_PO_4_, and 50 mL of Na_2_HPO_4_) (Fisher Scientific, Loughborough, UK). Pancreatin and pepsin were bought from Sigma Aldrich, UK for addition to the simulated intestinal fluid and simulated gastric fluid, respectively.

### 2.2. Model Bacterium and Bacteriophage

*E. coli* strain EV36, which is a K12/K1 hybrid developed by conjugation of Hfr kps + strain [25], was kindly provided by Dr. Eric R. Vimr. This strain is susceptible to K1-specific phages, including K1F and K1-5 [26]. Phage K1F was kindly provided by Dr. Dean Scholl (to Dr. Sagona) and is a T7-like phage first isolated from sewage in 1984 [27]. *E. coli* strain EV36 cells were made electrocompetent and were transformed with an RFP plasmid according to a published protocol [28].

*E. coli* strain EV36-RFP cells were cultured in a Luria–Bertani (LB) medium (Oxoid, Ltd., Basingstoke, UK) with the addition of 0.5 mM IPTG and 10 mg/mL Ampicillin sodium salt (Sigma Aldrich, Gillingham, UK).

Phage K1F was propagated on *E. coli* strain EV36-RFP. Phage stocks were propagated by growing a fresh culture of *E. coli* strain EV36-RFP in an incubator shaker at 37 °C until the OD_550_ reached 0.2. Subsequently, phage K1F was added at a Multiplicity of Infection (MOI) of 0.01. Following complete lysis of the bacteria, the culture was centrifuged for 15 min at 2000× *g* and the supernatant was filtered through a 0.2-µm filter (Millipore, Watford, UK). The phage stock was stored at 4 °C until further use.

The double-layer agar method was used for plaque assays, as described by Mahony et al. [29] and Goh at al. [30]. Briefly, 10 µL of overnight culture of *E. coli* strain EV36-RFP was added to a 50:50 mixture of LB soft agar 0.9% (*w*/*v)* and salt mixture (0.4 M MgCl_2_ and 0.1 M CaCl_2_; Oxoid Ltd. Basingstoke, UK). This was poured onto LB agar plates and set under a laminar flow hood. Ten µL of phage solution was serially diluted ten-fold in LB over an 8-log dilution range 10^−1^ through 10^−8^. Each dilution was spotted in triplicate and incubated at 37 °C overnight. The next day, the number of phage plaques in each spot were enumerated. In a similar manner, for bacterial growth, serial dilutions of bacterial cultures were spotted on LB agar and incubated overnight at 37 °C. Bacteria concentration was determined by counting colonies and expressed as a colony forming unit (CFU/mL).

### 2.3. Free Phage Sensitivity at Different pH Values

Simulated gastric fluid (SGF) was used to test phage sensitivity to different pH values. SGF formulation contained 0.2 M NaCl with pepsin at 3.2 mg/mL. Solution pH 2, 2.5, and 3 were adjusted using 0.1 M HCl. For simulated intestinal fluid (SIF) at pH 4 to 7, 0.2 M Sorensen’s buffer was used with the addition of 10 mg/mL pancreatin. Time points were taken at 0 min, 30 min, 1 h, 3 h, 5 h, and 24 h. For phages exposed to pH 2 and 2.5, exposure time points were also taken every minute for the first 10 min. A 10-µL sample was removed at each time point and serially diluted 10-fold in LB to 10^−8^ as described above. LB broth was used as a positive control.

### 2.4. Preparation of Bacteriophage-Containing Water-in-Oil (W/O) Emulsion

The dispersed phase (aqueous phase containing the bacteriophages mixed in a pH-responsive polymer formulation) was prepared by dissolving Eudragit^®^ S100 powder (final working concentration of 10% (*w*/*v*) in ultrapure water. Typically, 4 mL of 4 M NaOH solution was added to 36 mL of deionised water and 4 g of S100 powder was added to this solution in 100-mL Duran bottles equipped with a magnetic stirrer bar to aid stirring. The solution was left stirring overnight at 60 °C or until the solution was clear, with complete dissolution of the S100 powder. The resulting solution pH was typically between pH 6.5–7. To this solution, 0.4 g of sodium alginate powder was dissolved by stirring overnight at 60 °C. Ten mL of phage stock with titre ~10^9^ PFU/mL in an SM buffer (Trizma base (50 mM, Sigma Aldrich, Gillingham, UK), NaCl (100 mM, Fisher Scientific, Loughborough, UK), MgSO_4_·7H_2_O (8 mM, Fisher Scientific, Loughborough, UK), and 5 M HCl (~10 mL added per litre to adjust to pH 7.5)) was concentrated ten-fold using 100 kDa Amicon Ultrafiltration centrifuge tubes (Millipore, Watford, UK). One mL of concentrated phage stock was added to the cooled S100/alginate solution, resulting in a final phage titre of ~10^9^ PFU/mL in the final formulation used to make the microparticles. The continuous (oil) phase for the water-in-oil (W/O) emulsion was prepared by dissolving 5% (*w*/*w*) of PGPR in a mixture of Miglyol 840 and castor oil (9:1 by volume).

**Figure 1:**
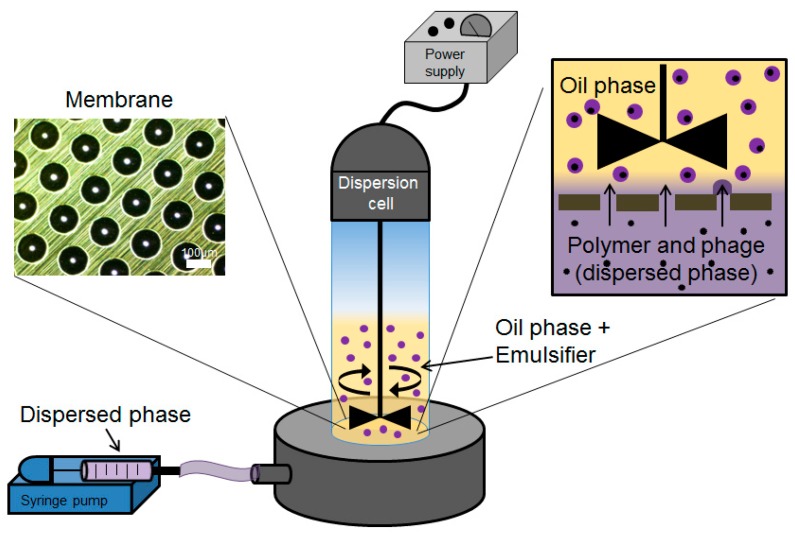
Schematic representation of the membrane emulsification system used for microencapsulation of bacteriophages.

Phage-containing S100/alginate droplets were prepared as a W/O emulsion using a batch membrane emulsification dispersion cell (Micropore Technologies Ltd., Redcar, UK). The dispersed phase was introduced into the bottom of the cylindrical glass emulsion chamber (working volume ~100 mL) using a syringe pump (Harvard Apparatus, Cambourne, UK). The base of the cell was equipped with a flat stainless-steel membrane having circular (3.3 cm diameter), 40-µm micropore arrays (Figure 1). The flow rate of the aqueous phase was controlled at 25 mL/h, equating to a flux of 29 l/m^2^ h. The glass cell carried the continuous oil phase above the membrane surface. Controlled shear was provided at the membrane surface using a paddle-blade stirrer operated by a 12-V DC motor with the rotation rate set at 250 rpm. Shear at the membrane surface is necessary for droplet detachment from the membrane pore resulting in a W/O emulsion. The dispersed phase was injected through uniformly spaced membrane pores, which were dispersed uniformly over the membrane surface. The stainless-steel membranes were purchased from Micropore Technologies Ltd. (Teesside, UK). They were pretreated with a hydrophobic silane coating by immersing the membrane for 10 min in 1H, 1H, 2H, 2H-Perfluorodecyltriethoxysilane (Sigma Aldrich, Gillingham, UK) prior to use. The stainless-steel membrane with pore size 40 µm had an effective surface area of 8.54 cm^2^, with pores spaced at a distance of 200 µm. The continuous phase volume used was 50 mL for every 5 mL of dispersed phase introduced into the glass cell.

### 2.5. Preparation and Characterisation of S100/Alginate Microparticles

Fifty-five mL of the W/O emulsion was transferred into 60 mL of the acidified-oil phase in a 150-mL glass vessel. The vessel was equipped with an overhead-stirrer with a three-bladed marine impeller (to provide good axial mixing), which was operated at a low agitation rate ~150 rpm (to avoid droplet breakup and settling). The acidified oil consisted of a 9:1 mixture of miglyol to castor oil with 5% (*v*/*v*) PGPR and 0.05 M toluenesulfonic acid (TSA). The droplets were left to crosslink for 1 h and then allowed to settle (overhead stirrer was turned off). The supernatant was discarded, and any residual miglyol was washed out using 99.9% analytical grade hexane (Sigma Aldrich, Gillingham, UK). The TSA-crosslinked microparticles were resuspended in 0.1 M calcium chloride (CaCl_2_) with 2% (*v*/*v*) Tween 20, and the solution was pre-acidified at pH 3 (using 0.1 M HCl). Microparticles were left in suspension to crosslink the alginate for 2 h. The crosslinked particles were subsequently allowed to settle, the CaCl_2_ solution was removed, and the microparticles were washed with 2% (*v*/*v*) Tween 20 acidified at pH 3. The gelled microparticles were stored at 4 °C in sealed, 15-mL falcon tubes.

Photographs of the droplets and particles were taken with an optical microscope (Nikon Eclipse E200, Kingston-Upon-Thames, UK) using a ×4 magnification objective lens. The particle sizes for the W/O emulsion and the gelled microparticles were measured using a Coulter LS series 130 (Beckman Coulter Inc., High Wycombe, USA) instrument employing a Fraunhofer optical model for data regression.

### 2.6. Sample Preparation for the Ion Microscopy

Critical point drying (CPD) and freeze-drying methods were used to prepare the hydrogel samples for ion microscopy. In the CPD-method, the silicon substrate (Tedpella, Redding, CA, USA) was incubated for 5 min in poly-l-lysine (150–300 kMW, Sigma-Aldrich, Hamburg, Germany), washed three times with ion-exchanged water, and allowed to dry under ambient conditions (room temperature and atmospheric pressure). Using a spatula, hydrogel microparticles were added to 500 µl acetate buffer (50 mM, pH 5, 20 mM CaCl_2_) and vortexed. The solution was pipetted over the lysine coated substrate, and the microparticles were allowed to adhere for about 1 h at 4 °C and, after that, were fixed overnight with 2.5% glutaraldehyde in 0.1 M acetate buffer (pH 5) at 4 °C. After fixing, the sample was washed two times with 0.1 M acetate buffer (pH 5), stained with 1% OsO_4_ for 30 min, and then washed three times with the buffer. Samples were dehydrated in ethanol (EtOH) using a series of steps with increasing EtOH concentration: 50, 70, 90, 95, and 2 × 99.5%, 15 min each. Dehydrated samples were dried to ambient conditions with CPD (Leica CPD 300) using 16 exchange cycles. The dried sample was attached to the metal stub with carbon tape prior to microscopy. In the freeze-drying method, gelled microparticles were frozen on filter paper (0.2-µm pore size, Millipore Ltd., Watford, UK) at −20 °C overnight. The particles were freeze dried (VirTis Wizard 2.0, SP Scientific, New York, NY, USA) for 24 h at 50 Pa pressure and −20 °C. Dried powder was applied directly on the carbon tape, which was attached to the sample stub.

### 2.7. Ion Microscopy

To analyse the morphology of the hydrogel particles, both freeze-dried and critical-point-dried hydrogels were examined with ion microscopy. Zeiss Orion NanoFab (University of Jyväskylä) with He^+^ beam and acceleration voltage 35 kV, 0.20 pA current, 32 line averages, and 1 µs dwell time was used for He^+^ imaging. For cutting, an about 20-pA Ne^+^ beam with 10 kV acceleration voltage was used. Milling was carried out using a 45 degrees tilted angle by setting the reduced raster scan rectangle over the area to be removed and scanning until the material disappeared. After cutting, the sample stage was rotated 180° and the cross section was imaged with a He^+^ beam. Flood gun charge compensation was used during both milling and imaging.

### 2.8. Encapsulated Phage Release from Microparticles in SIF Following Exposure to SGF

Typically, 1 g of hydrogel microparticles was added to prewarmed 10 mL SIF and shaken in a temperature-controlled incubator shaker (Certomat, Sartorius, UK) at 120 rpm and 37 °C. For SGF exposure, the microparticles were first added to 10 mL SGF and left shaking for an exposure period of 2 h at 37 °C. Particles were centrifuged at 2000× *g* for 10 min and resuspended in 10 mL SIF (pH adjusted to 5, 6, or 7) until dissolution of the particles was complete. Spot assays were used for enumeration of the released phage by removing 10 µL of the sample at hourly intervals, by serially diluting in LB broth, and by spotting on a host bacterial lawn.

### 2.9. Storage Stability of Bacteriophages Encapsulated in the Microparticles

Microparticles were stored in screw-top bottles under controlled refrigerated conditions (4 °C) for a period of 4 weeks. At specific time intervals, 0.1 g of microparticles was weighed and dissolved in SIF (as per the protocol outlined above). Phage release was enumerated using plaque assays.

### 2.10. In Vitro Assay to Measure Phage Release and E. coli Killing Dynamics

The effect of encapsulated phage (EK1F) and free phages in killing bacterial was investigated. Bacteria were grown from a single colony until log phase was reached at 0.2 (O.D. 550) (approximately 10^8^ CFU/mL). *E. coli* strain EV36 bacteria were grown either in LB or cell culture media (Leibovitz), as described previously [28]. Two mL of bacteria (at 0.2 OD) were added to 24-well tissue-culture plates. Twenty µL of free phage (non-encapsulated) was added at different concentrations (resulting in working concentrations of 10^2^, 10^4^, 10^6^, and 10^8^ PFU/mL). Twenty µL of encapsulated phages (EK1F) were also added to separate wells containing 2 mL of bacterial culture. Encapsulated phages were tested either without acid exposure or after exposure to SGF (pH 2) for 2 h. Negative controls contained 20 µL of EK1F in LB media or cell culture media without bacteria. Positive controls contained bacteria only. After phage/EK1F addition, the samples were withdrawn at hourly intervals up to 5 h post phage/EK1F addition. At each time point, 1 mL of sample was removed to record the O.D. One mL of the sample was centrifuged at 2000× *g* to remove infected cells and to measure phage titre using plaque assay.

### 2.11. E. coli Killing Using Encapsulated Phages in an Epithelial Cell Culture Assay

The human urinary bladder epithelial cell line (ATCC^®^ HTB-4^™^) was acquired from LGC Standards (UK). T24 cells were cultured in uncoated T75 flasks containing McCoy’s 5A (Modified) medium (Gibco, MA, USA) supplemented with 10% *v/v* Foetal Bovine Serum (FBS) (Labtech International, UK) and 1% *v/v* Penicillin-Streptomycin and were maintained in 5% CO_2_ under a humidified atmosphere, as described previously [28]. For the live microscopy experiments, the T24 cells were seeded onto uncoated 35-mm glass-bottom microscope dishes (ThermoFisher Scientific, UK) at a density of approximately 4 × 10^4^ cells/cm^2^ in McCoy’s 5A (Modified) medium (supplemented with 10% *v/v* FBS only) and were allowed to settle for 24 h. The culture media were aspirated and replaced with Leibovitz medium (Lonza, Switzerland) that sustains cell viability in the absence of CO_2_ equilibrium. While maintaining the confluent cultures at 37 °C, the cultures were incubated with *E. coli* strain EV36-RFP at OD 600 nm of approximately 0.4 to 0.6 (~10^8^ CFU/mL), which was added to 1 mL of Leibovitz media for 2 h. Controls included bacteria left to grow with the addition of LB (20 µL) without phages. Free phages at different titres (10^2^, 10^4^, 10^6^, and 10^8^ PFU/mL) (volume 20 µL) were added to wells as positive controls. Microparticles with phage K1F (EK1F) were exposed to pH 2 for 2 h before they (20 µL, ~10 mg) were added to infected epithelial cells. In the last 20 min of infection, NucBlue^®^ Live (ThermoFisher Scientific, UK) was added as a stain for the nucleus. The samples were then visualized under an Andor/Nikon Spinning Disk Confocal Laser Microscope. Imaging was taken at hourly intervals (each sample was staggered accordingly). During imaging, the temperature was maintained at 37 °C.

For the experiments with fixed cells on the confocal microscope, T24 cells were seeded onto uncoated 22 × 22 mm coverslips in 6-well plates at a density of 4 × 10^4^ cells/cm^2^ and were allowed to settle for 24 h. The cultures were fixed in 4% paraformaldehyde (PFA) (ThermoFisher Scientific, UK), permeabilised in ice-cold PEM (PIPES, EGTA, Magnesium) buffer with 0.05% Saponin, and quenched with 50 mM NH_4_Cl in PBS (Phosphate-Buffered Saline). A wash step with PBS was performed between each step, as described previously [28]. In order to visualize the periphery of the cells, an actin filament stain, Phalloidin CF680R Conjugate (Biotium, CA, USA), was used at a concentration of 5 ug/mL. Finally, the stained cells were mounted on microscope slides using Flouroshield Mounting Medium (Abcam, UK) containing DAPI nuclear stain. All fixed cells were imaged using the Zeiss LSM 880 confocal microscope with Airyscan.

### 2.12. Statistical Analysis of Results

Minitab version 18 (Minitab UK Ltd, Coventry, UK) was used for carrying out statistical analysis of the data. Sample means were compared using two-sample t-tests (*n* = 3) with reporting of *p* < 0.05 as statistically significant. For multiple tests, the alpha value was adjusted using Bonferroni correction.

## 3. Results

### 3.1. Production of Microencapsulated Phages Using Membrane Emulsification

**Figure 2:**
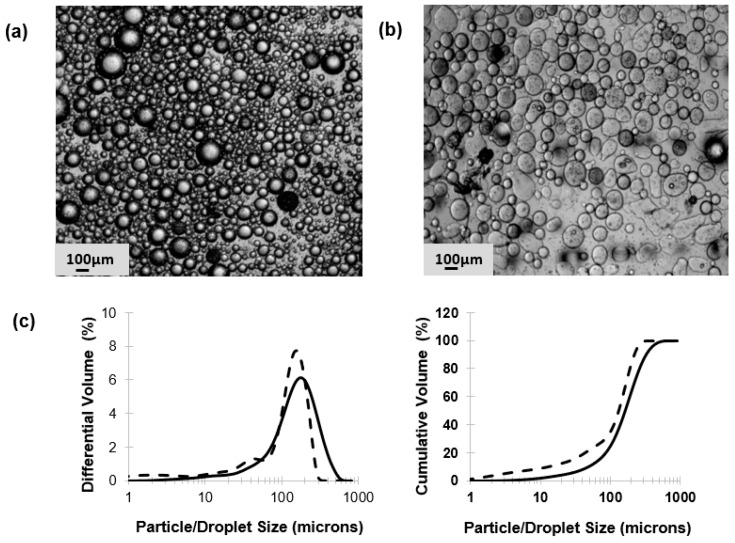
Optical images of water-in-oil (W/O) emulsion droplets, crosslinked microparticles, and size distribution of phage-containing emulsion droplets and microparticles: (**a**) water-in-oil emulsion, (**b**) microparticles produced from W/O emulsions following toluenesulfonic acid (TSA) and CaCl_2_ crosslinking, and (**c**) droplet and particle size distributions (solid lines—droplets; dashed lines—particles).

Membrane emulsification was used to prepare phages encapsulated in a W/O emulsion with the volume mean droplet size d50 of ~200 µm (Figure 2). The size distribution of the W/O emulsion indicated the presence of smaller droplets that contributed around 20% of the total volume fraction (Figure 2c). The mean droplet size could easily be varied by changing the shear rate (by varying the rotation speed of the impeller), the membrane pore size, and the viscosity of the continuous phase (data not shown). Conditions were selected to yield droplets in the 100–300 µm range. Gelation of the drops by crosslinking with TSA and calcium chloride resulted in distinct gelled solid microspheres. A slight reduction in the final particle size was noted compared with the primary W/O emulsion, d50 reducing to just over 100 µm (Figure 2b). The hydrogel microparticles easily passed through a 15 G oral gavage needle, indicating they were suitable for oral administration in mice or rats.

### 3.2. Microstructure of pH-Responsive Hydrogel Microparticles

**Figure 3:**
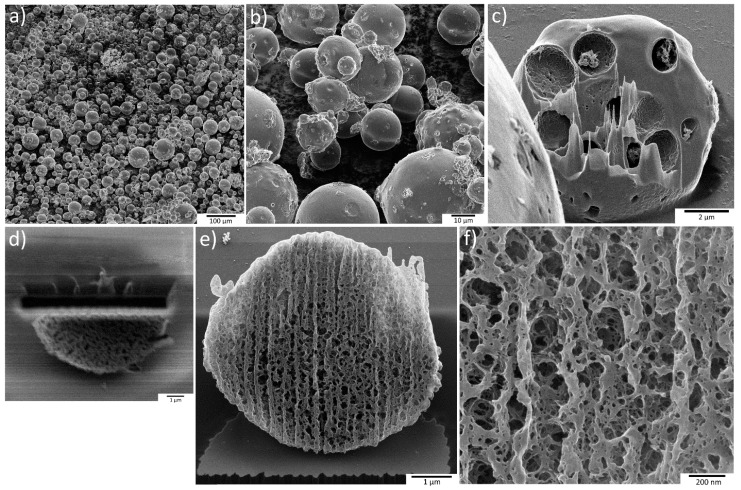
HIM images of freeze-dried and CPD-dried microcapsules: (**a**) Freeze-dried sample with a 1-mm field of view and (**b**) with higher magnification, and (**c**) a microcapsule having a particle size of about 10 µm was cut in half using a Ne^+^ beam and, after a 180° rotation, was imaged with He^+^. (**d**) A CPD-dried microparticle was also milled with the Ne^+^ beam. (**e**) After 180° rotation, the cut surface was imaged with He^+^. (**f**) A higher magnification image of the cross section shows the internal porous matrix of the polymer.

The morphology of the microparticles was found to depend upon the sample preparation method (Figure 3). Freeze-dried microparticles appeared as spheres with a smooth and uniform surface (Figure 3a,b). Cutting with Ne^+^ allowed visualization of the internal structure of the freeze-dried microparticles, which were found to be porous (Figure 3c). CPD-dried microparticles displayed a sponge-like surface (Figure 3d). The internal structure was found to be porous with an interconnected network of much smaller pores compared to the freeze-dried sample (Figure 3e,f).

### 3.3. Effect of Acid Exposure on Free Phage and Encapsulated Phage Viability

**Figure 4:**
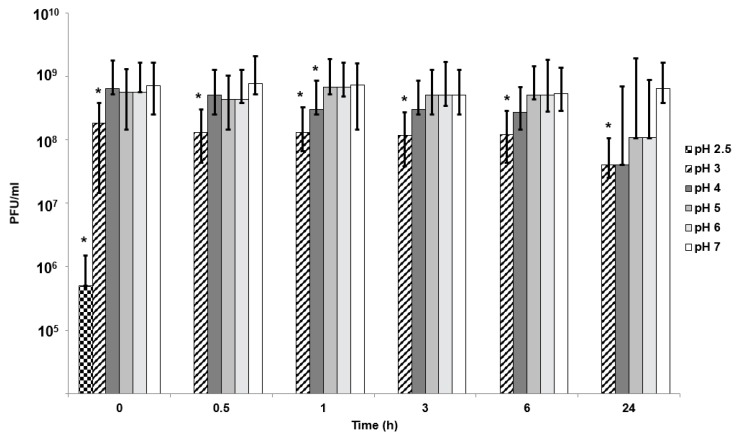
Effect of pH on free phage viability in 0.2 M NaCl solution. *Significantly different phage titres using a 2-sample *t*-test at each condition compared with phages exposed to pH 7 at the corresponding time point. Time point 0 h denotes the time between 0–10 min for phages exposed to all pH values except pH 2.5, where the phage titre reduced rapidly, and data is plotted 10 s post exposure.

*E. coli*-phage K1F lost nearly all activity within 30 min of exposure to pH 2.5 with a modest reduction in phage titre upon exposure to pH 3 (Figure 4). Unencapsulated phages were stable upon exposure to solutions with pH adjusted to pH 4 and above over a 24 h exposure period.

**Figure 5:**
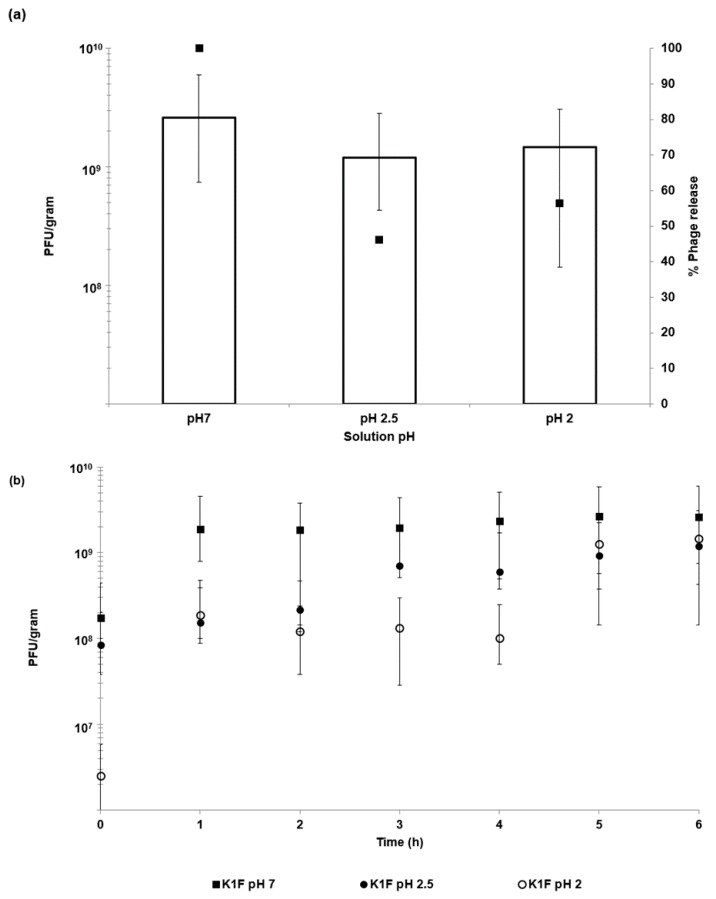
Release of encapsulated bacteriophages from ES100/Alg (EK1F) microparticles: (**a**) Total phage release from EK1F microparticles after 6 h exposure to Simulated Intestinal Fluid (SIF) at pH 7 with and without exposure to Simulated Gastric Fluid (SGF) at pH 2 and pH 2.5 (exposure to SGF for 2 h). The black squares represent the % phage release compared to microparticles not exposed to SGF (pH 7). (**b**) Phage release kinetics from EK1F microparticles over a 6 h exposure period to SIF at pH 7 without and with exposure to SGF at pH 2 and pH 2.5.

Acid stability of encapsulated K1F phages was assessed by exposing the encapsulated phages to SGF with pH adjusted to pH 2 and pH 2.5 (exposure period 6 h). Thereafter, the microparticles were exposed to SIF (pH 7). There was no statistically significant difference in the amount of phages recovered post exposure to pH 2 and pH 2.5 compared with virgin microparticles exposed only to SIF (Figure 5a). Thus, the microparticles afforded complete protection to the encapsulated phages from SGF and released the phages upon subsequent exposure to SIF at pH 7 (Figure 5b). Measurement of the phage release kinetics indicated that most of the encapsulated bacteriophages were released within a 2 h period post SIF exposure for the virgin microparticles; however, the acid exposed microparticles showed around 50% of the encapsulated phages were released after 2 h (Figure 5a). Almost full phage release took ~5 h for the acid exposed microparticles with the amount of viable phages no different to those released from the virgin microparticles (not exposed to SGF).

**Figure 6:**
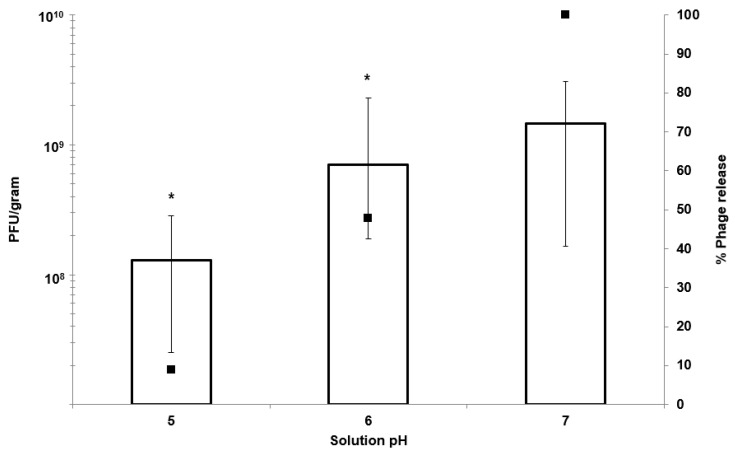
Release of encapsulated bacteriophages from EK1F microparticles: Phage release (PFU g^−1^) after 5 h from EK1F microparticles in Simulated Intestinal Fluid (SIF) at different pH values following prior exposure of microparticles to Simulated Gastric Fluid (SGF) at pH 2 (2 h exposure to SGF). The black squares represent the % phage release. *Significantly different phage titres (*p* < 0.05) for a 2-sample *t*-test with each sample compared with phage release from EK1F exposed to pH 7.

Phage-encapsulated microparticles that were previously exposed to SGF (exposure for 2 h, at pH 2) were subsequently tested for phage release at different pH (pH 5, 6, and 7). Less than 10% of the total encapsulated phage dose was released from the microparticles at pH 5 after 2 h (Figure 6). This increased to around 40% at pH 6 and complete release at pH 7 indicating encapsulated phages would be released in areas of the gastrointestinal tract where the pH was 6 or higher.

### 3.4. Dynamics of Bacteria Killing by Free Phage and Encapsulated Phage Using an In Vitro Assay

**Figure 7:**
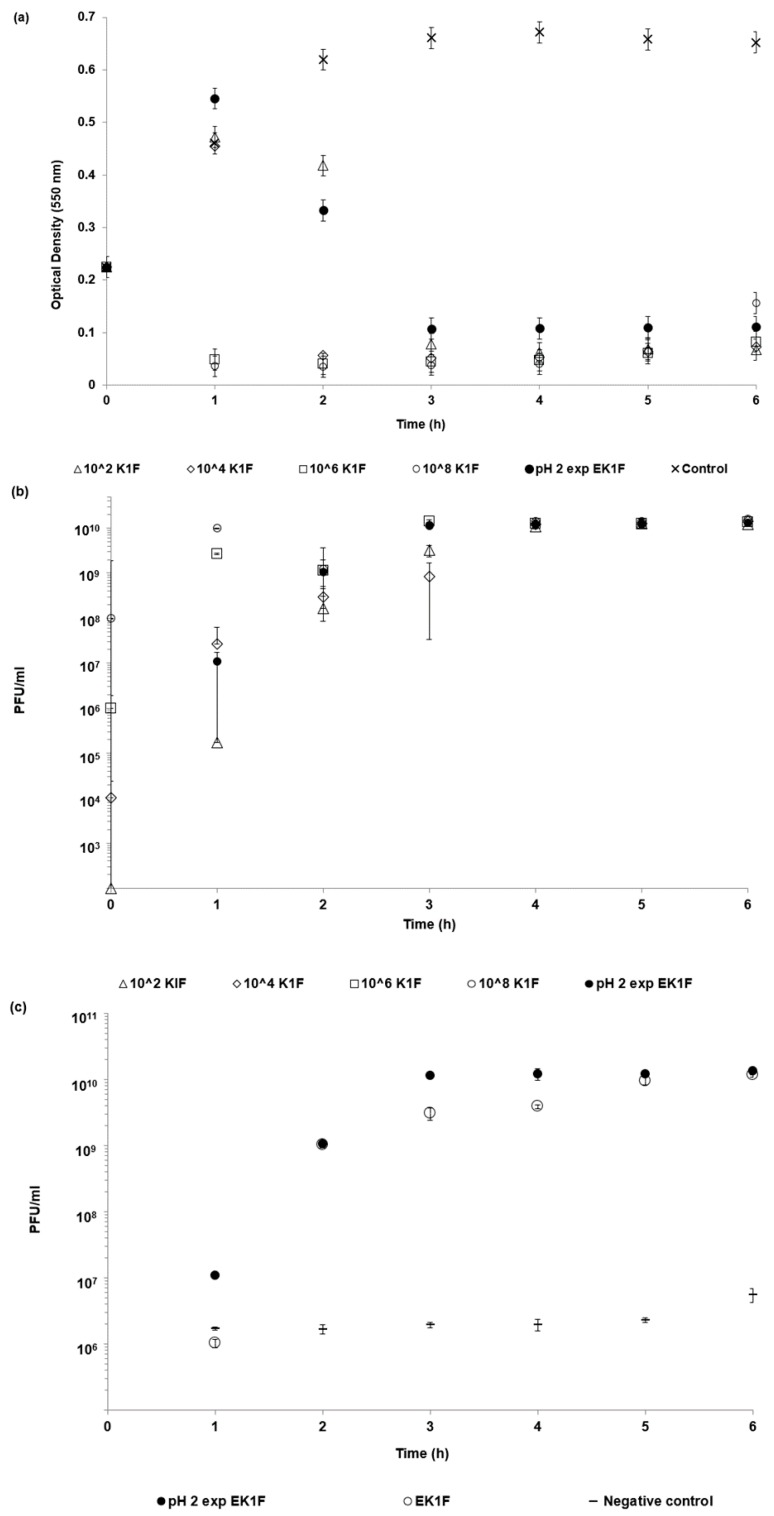
Dynamics of phage killing of *E. coli* strain EV36 bacteria with K1F bacteriophages: (**a**) Optical density curves showing bacteria killing in the presence of different concentrations of added phage, (**b**) phage amplification in the presence of *E. coli* strain EV36, and (**c**) comparison of the phage amplification of EK1F phages encapsulated in microparticles with and without exposure to SGF (2 h). Error bars represent one standard deviation.

The optical density of bacterial cultures was rapidly reduced (within 1 h) following the addition of free phages (not encapsulated) at doses of 10^6^ and 10^8^ PFU/mL (Figure 7a) as compared with negative controls (no added phage). Addition of free phages at lower doses of 10^2^ and 10^4^ PFU/mL resulted in continued increase in the optical density of the cultures for the first hour, followed by a decline thereafter; the sample dosed with 10^2^ PFU/mL of free phages took 3 h for the OD value to fall below 0.1. The OD profile for the SGF-exposed microencapsulated phages (EK1F) mimicked that of the 10^4^ PFU/mL dose. Phage amplification in cultures with low phage dose addition at 10^2^ and 10^4^ PFU/mL lagged those where doses of 10^6^ and 10^8^ PFU/mL had been added (Figure 7b). Addition of SGF-exposed EK1F microparticles resulted in in situ phage amplification mimicking the profiles for the low free phage dosed cultures. Phage amplification from virgin and SGF-exposed microparticles in the bacterial cultures showed no significant difference in the time it took for the released phages to amplify (Figure 7c). Negative controls (absence of bacteria) showed release of a dose of 10^6^ PFU of phages from the virgin microparticles after 1 h of exposure to the culture media.

### 3.5. Dynamics of Bacteria Killing by Encapsulated Phage Using an In Vitro Epithelial Cell Assay Observed Using Live and Confocal Microscopy

**Figure 8:**
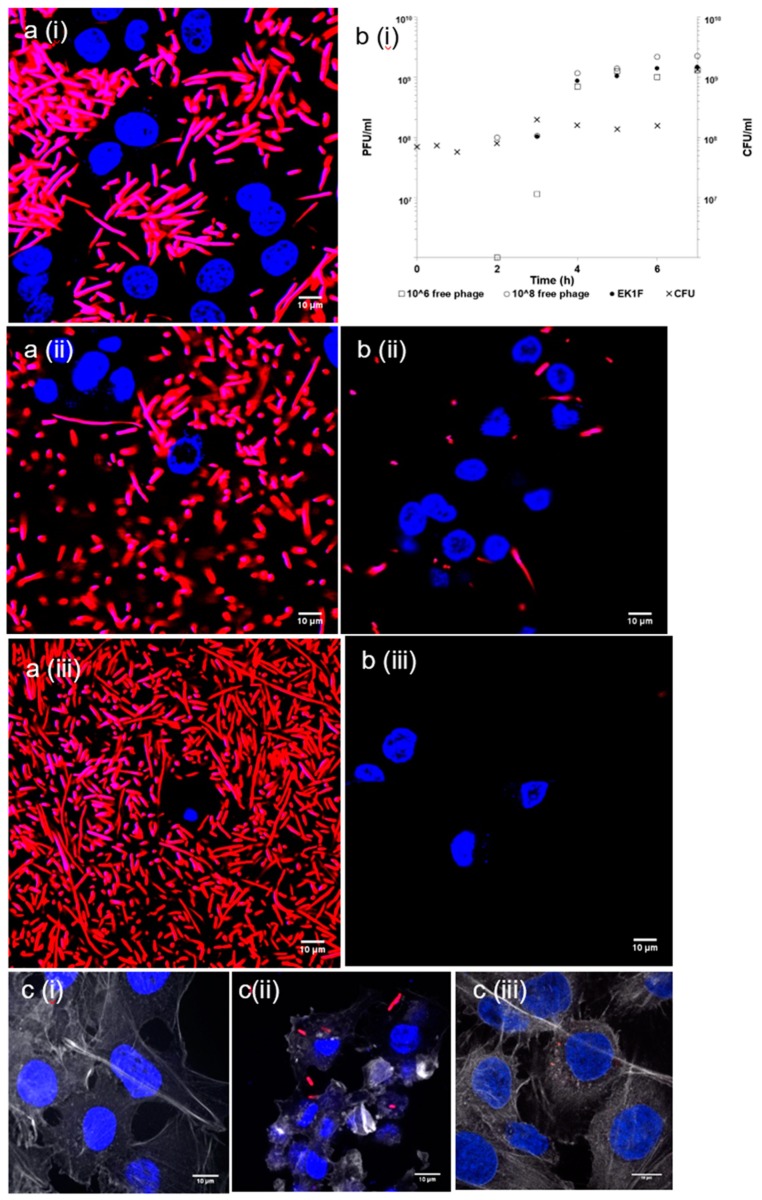
Live and confocal microscopy images of *E.coli* strain EV36-RFP cells (red) treated with microencapsulated EK1F in the presence of epithelial cells (blue): (**a**) Controls without phages (i) 2 h, (ii) 3 h, and (iii) 5 h following incubation of epithelial cells with *E. coli* strain EV36; (**b**) (i) phage amplification of free phages and EK1F in the presence of *E.coli* strain EV36, and live microscopy images of EK1F treated samples at (ii) 3 h and (iii) 5 h following incubation of epithelial cells with *E.coli* strain EV36; (**c**) (i) control epithelial cells not exposed to *E.coli* strain EV36, (ii) damaged actin visible for epithelial cells exposed to EV36 with no phage treatment, and (iii) the condition of actin of epithelial cells exposed to *E.coli* strain EV36 and treated with EK1F.

Actively growing *E. coli* strain EV36 bacterial cells could be visualised using live and confocal microscopy in the presence of epithelial cells (Figure 8, highlighted by the pink/red fluorescent rods). Epithelial cells growing in the presence of actively replicating bacteria showed signs of stress, including shrinking cell nucleus morphology and cell death (Figure 8a i–iii) and visible damage to the actin cytoskeleton (Figure 8c ii). Actin provides support for the cell shape, cell division, and other cellular processes. Epithelial cells treated with SGF-exposed encapsulated phages (phage dose 10^8^ PFU/mL) showed clear differences compared with untreated controls (where no phage had been added but bacteria were actively replicating). Phage amplification in the presence of epithelial cells was similar to free phages added at an equivalent dose (Figure 8b i). The concentration of bacterial cells was significantly reduced by the addition of encapsulated phages (Figure 8b ii,iii). The morphology of the epithelial cell nucleus was considerably better, and taut microtubules could be seen (Figure 8c iii) compared with collapsed ones for the untreated controls (Figure 8c ii). Under these conditions, the human cells were considerably healthier and homogeneous compared to control, uninfected cells. The nucleus of healthy cells (Figure 8c i,iii) had a clear round or oval shape and were large. The nuclei of infected cells not treated with phages lost their round shape and appeared considerably smaller. The perimeter of 3 nuclei per image were selected (Figure S1), and corresponding areas for the nuclei were calculated. The area of the nucleus of infected cells was around 50% smaller compared with healthy controls (Table S2).

### 3.6. Storage Stability of Encapsulated Phage

**Figure 9:**
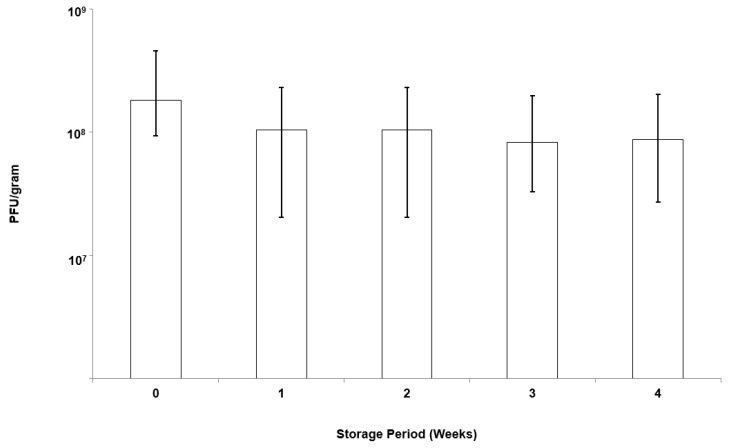
Storage stability of K1F phages in EK1F microparticles refrigerated at 4 °C: Phage titre was evaluated by exposing microparticles to SIF.

EK1F microparticles were stored over a course of 4 weeks under refrigerated conditions (at 4 °C). There was no statistical change in the K1F phage titre, which remained constant at ~10^8^ PFU g^−1^ over the storage period (*p* < 0.05) (Figure 9 and Table S1).

## 4. Discussion

The motivation behind the microencapsulation of phages in solid oral dosage forms stems from the need to protect bacteriophages from gastric acidity and to ensure delivery of high phage doses to specific intestinal compartments at the site of infection, such as the ileum or colon. Targeted delivery of viable phages avoids issues such as a reduction in phage titres due to dilution effects *en route* to the infection site in the lower gastrointestinal tract. Small microparticles (size less than 0.5 mm) are particularly useful for phage delivery via oral gavage for routine preclinical in vivo testing in small animals such as mice and rats. Previous published studies suggest that particles around 100 µm are well suited in terms of protecting bacteriophages from gastric acidity typically encountered in the stomach environment [23,31]. We have previously demonstrated the potential of microfluidic fabrication processes for phage microencapsulation in uniform small pH-responsive microparticles (mean size ~100 µm) containing an encapsulated *Salmonella Myoviridae* Felix O1 phage [31]. Microfluidic single droplet generation units suffer from low throughputs with typical production rates of less than 1 mL/h. Here, we have demonstrated the scalability afforded by the process of membrane emulsification whereby phages may be encapsulated under low shear rate conditions resulting in near 100% phage encapsulation efficiency and preparation of large industrial quantities of uniform small microcapsules (Figure 2). The process can be scaled-up based on the membrane area, e.g., a typical tubular membrane module (50-cm tube length and 1-cm diameter) would produce 0.5 L/h of microparticles operating under the conditions used in the present study. Tube bundles in a shell-and-tube arrangement would allow considerably higher production rates. The membrane emulsification process itself is generic in terms of production of W/O emulsions containing different enteric phages. The process is highly flexible such that different formulations can be used and microparticles with phage-release properties can be tailored for specific applications using different stimuli-responsive polymers. Phages are known to be sensitive to chemical and physical stresses; these include pH [32], temperature [33], exposure to organic solvents [34,35], shear [33,36], and ionic strength [37]. A number of studies have previously shown that particle size is an important factor affecting phage protection from SGF for acid permeable beads [31,38]. We have shown that Eudragit ES100 co-formulated with medium viscosity alginate in 100-µm microcapsules protected the phages exposed to SGF at pH 2 for an exposure period of 2 h (Figure 5). We have also shown that, when using an in vitro bacterial growth assay, phage-mediated rapid killing of actively growing bacteria requires high titres of viable phages to arrest bacterial growth in a timely fashion (Figure 7). Furthermore, we have demonstrated the suitability of using membrane emulsification to prepare *E. coli*-specific K1F phages encapsulated in pH-responsive microcapsules suitable for gastrointestinal applications (Figure 5). The internal microstructure of the hydrogel beads was shown to consist of a matrix-like polymer network. In all likelihood, the polymer microstructure is swollen in a hydrated state yielding solid core microparticles, which afforded the phages protection from the external gastric acid environment (Figure 3). It was not possible to image encapsulated bacteriophage particles within the internal structure, suggesting that most probably these were buried within the hydrogel matrix. Preparation methods clearly affected visualization of the inner structure of the microparticles. CPD-dried samples most probably represent the wet hydrogel microstructure more accurately because CPD-drying avoids all phase boundaries during the drying process. Previously, it has been shown that freeze-drying modifies the gel matrix structure towards bigger pores [39].

In vitro phage stability experiments where phages (encapsulated and free phages) are exposed to different acidic pH encountered in vivo may be a useful predictor of in vivo phage survival and a prerequisite during formulation development prior to testing in animal models. Unencapsulated bacteriophages were highly sensitive to acidic pH exposure with complete loss of viable phages within 10 min at pH 2.5 (Figure 4). The loss of unencapsulated phage activity upon exposure to an acidic environment highlights the need for encapsulation in order to deliver controlled high doses of viable phages to the infected gut using oral dosage forms. Phage encapsulation in the ES100/alginate microparticles protected them from acid damage at pH 2 (Figure 5). The combination of small particle size (Figure 2) and pH-responsive character of the ES100/alginate microcapsules resulted in release of encapsulated K1F phages within the first hour upon exposure to pH 7 (Figure 5b). Ten percent of the encapsulated phage dose was released at pH 5, whereas 50% of it was released at pH 6 after 2 h of exposure to SIF. Complete phage release and complete dissolution of microparticles occurred within 1 h upon exposure to pH 7 (Figure 5b). It took 1 h longer for SGF-exposed EK1F phages to amplify to levels reached in bacterial cultures compared with a free phage dose of 10^6^ PFU/mL (Figure 7b). This delay may be attributed to slower release kinetics of SGF-exposed EK1F phages (Figure 5b). The encapsulated phages were not released immediately upon addition of the microparticles to the bacterial cultures; therefore, it took extra time for the phage titre to reach levels similar to an equivalent free phage dose. Previous published research on phage encapsulation employing large microparticles (~1 mm) reported slower sustained-release kinetics for phages encapsulated in alginate microcapsules [19,22]. Colom et al. [23] reported faster release from small alginate microparticles containing CaCO_3_ as antacid (mean size ~100 µm). However, in that particular study, exposure to simulated gastric fluid (pH 2.8 for 60 min) resulted in between 2 and 3 log reduction in *Salmonella* phage titre, suggesting that even with the addition of CaCO_3_, phages were highly susceptible to SGF [23]. The ES100/alginate formulation used in the present study for similarly sized small microparticles showed considerably better acid protection and released phages in response to a pH-trigger.

A number of in vivo animal studies have demonstrated dose-dependent phage therapy outcomes for the treatment of bacterial infections, with high doses resulting in better clinical results [40,41,42,43]. Controlling the phage dose delivered at the site of infection and controlling the timing of the delivery are important considerations [14]. Careful formulation and encapsulation of phages in uniform-sized microcapsules may facilitate better control over the phage release dynamics. This would allow control over the accurate delivery of high concentrations of phages and effective killing of actively growing *E. coli* strain EV36 cells as shown using an in vitro assay (Figure 7). Even after exposure of encapsulated K1F phages in the microparticles to simulated gastric fluid at pH 2 for 2 h, the released phage dose was unchanged: ~10^9^ PFU g^−1^ of microparticles (Figure 5). In humans the mean residence time for gastric emptying is typically less than 3 h for both pre-fed and fasted states [44]. In the absence of phage encapsulation, a loss in phage titre due to stomach acidity exposure would result in a low dose of phages reaching the site of infection. This may potentially result in a lack of in situ phage amplification in the presence of low concentrations of bacteria during infection [14].

Phage delivery systems and their formulation will impact phage bioavailability at the site of infection. Constituents of the formulation should not be toxic to the host (human) cells. Bacterial killing by K1F phages released from the microcapsules in the presence of epithelial cells suggested that the phages retained potency at levels similar to free phages at the same dose levels (Figure 8). Confocal and live microscopy images provided visual confirmation that phages released from microcapsules arrested bacterial damage to the epithelial cell actin cytoskeleton and significantly decreased the number of bacteria in the human cell environment compared with controls containing bacteria but not treated with phages (Figure 8). Furthermore, the release of encapsulated phages was not hindered by the microparticles interacting with epithelial cells. These in vitro results are encouraging in the development of phage oral dosage formulations as a precursor to future evaluation in animal systems such as mice or rats.

## 5. Conclusions

We have demonstrated the suitability of using membrane emulsification for phage microencapsulation to produce stimuli-responsive controlled release microparticles. The process is scalable and compatible with cGMP manufacturing and could have significant impact in the field of phage therapy. The controlled release formulations developed in the present study would allow precise delivery of high doses of enteric phages at the site of infection in the GIT with phage release triggered by a change in environmental pH. Other triggers such as the presence of certain enzymes or virulence factors should be explored in future studies.

## Supplementary Materials

The following are available online at https://www.mdpi.com/1999-4923/11/9/475/s1, Figure S1: Measurement of cell nucleus perimeter using Fiji image processing software and calculation of nucleus area for each of the cells shown in Figure 8c. Table S1: Summary of microencapsulated EKF1 storage results. Table S2: Summary of calculated areas of each cell nucleus.

## References

[1] Huttner A., Harbarth S., Carlet J., Cosgrove S., Goossens H., Holmes A. (2013). Antimicrobial resistance: A global view from the 2013 World Healthcare-Associated Infections Forum. Antimicrob. Resist. Infect. Control.

[2] Merril C.R., Scholl D., Adhya S.L. (2003). The prospect for phage therapy in western medicine. Nat. Rev. Drug Discov..

[3] Freire-Moran L., Aronsson B., Manz C., Gyssens I.C., So A.D., Monnet D.L., Cars O. (2011). Critical shortage of new antibiotics in development against multidrug-resistant bacteria—Time to react is now. Drug Resist. Updat..

[4] Czaplewski L., Bax R., Clokie M., Dawson M., Fairhead H., A Fischetti V., Foster S., Gilmore B.F., Hancock R.E.W., Harper D. (2016). Alternatives to antibiotics—A pipeline portfolio review. Lancet Infect. Dis..

[5] Alisky J., Iczkowski K., Rapoport A., Troitsky N. (1998). Bacteriophages show promise as antimicrobial agents. J. Infect..

[6] Abedon S.T., Kuhl S.J., Blasdel B.G., Kutter E.M. (2011). Phage treatment of human infections. Bacteriophage.

[7] Allen H.K., Trachsel J., Looft T., Casey T.A. (2014). Finding alternatives to antibiotics. Ann. N. Y. Acad. Sci..

[8] Abedon S.T. (2009). Kinetics of phage-mediated biocontrol of bacteria. Foodborne Pathog. Dis..

[9] Bikard D., Euler C., Jiang W., Nussenzweig P.M., Gregory W., Duportet X., Fischetti V.A., Marraffini L.A. (2015). Development of sequence-specific antimicrobials based on programmable CRISPR-Cas nucleases. Nat. Biotechnol..

[10] Barrow P.A., Soothill J.S. (1997). Bacteriophage therapy and prophylaxis: rediscovery and renewed assessment of potential. Trends Microbiol..

[11] Galtier M., De Sordi L., Maura D., Arachchi H., Volant S., Dillies M.-A., Debarbieux L. (2016). Bacteriophages to reduce gut carriage of antibiotic resistant uropathogens with low impact on microbiota composition. Environ. Microbiol..

[12] Levin B.R., Bull J.J. (2004). Population and evolutionary dynamics of phage therapy. Nat. Rev. Microbiol..

[13] Viswanathan V.K., Hodges K., Hecht G. (2009). Enteric infection meets intestinal function: how bacterial pathogens cause diarrhoea. Nat. Rev. Microbiol..

[14] Alam Sarker S., Sultana S., Reuteler G., Moine D., Descombes P., Charton F., Bourdin G., McCallin S., Ngom-Bru C., Neville T. (2016). Oral Phage Therapy of Acute Bacterial Diarrhea With Two Coliphage Preparations: A Randomized Trial in Children From Bangladesh. EBioMedicine.

[15] Young R., Gill J.J. (2015). Phage therapy redux—What is to be done?. Science.

[16] Denou E., Bruttin A., Barretto C., Ngom-Bru C., Brüssow H., Zuber S. (2009). T4 phages against *Escherichia coli* diarrhea: Potential and problems. Virology.

[17] Ma Y.-H., Islam G.S., Wu Y., Sabour P.M., Chambers J.R., Wang Q., Wu S.X.Y., Griffiths M.W. (2016). Temporal distribution of encapsulated bacteriophages during passage through the chick gastrointestinal tract. Poult. Sci..

[18] Choinska-Pulit A., Mitula P., Sliwka P., Choi A., Wojciech Ł., Skaradzinska A. (2015). Bacteriophage encapsulation: Trends and potential applications a. Trends Food Sci. Technol..

[19] Ma Y., Pacan J.C., Wang Q., Xu Y., Huang X., Korenevsky A., Sabour P.M. (2008). Microencapsulation of Bacteriophage Felix O1 into Chitosan-Alginate Microspheres for Oral Delivery. Appl. Environ. Microbiol..

[20] Tang Z., Huang X., Baxi S., Chambers J.R., Sabour P.M., Wang Q. (2013). Whey protein improves survival and release characteristics of bacteriophage Felix O1 encapsulated in alginate microspheres. Food Res. Int..

[21] Slopek S., Durlakova I., Weber-Dąbrowska B., Kucharewicz-Krukowska A., Dabrowski M., Bisikewicz R. (1983). Results of bacteriophage treatment of suppurative bacterial infections. I. General evaluation of results. Arch. Immunol. Ther. Exp. (Warsz).

[22] Kim S., Jo A., Ahn J. (2015). Application of chitosan-alginate microspheres for the sustained release of bacteriophage in simulated gastrointestinal conditions. Int. J. Food Sci. Technol..

[23] Colom J., Cano-Sarabia M., Otero J., Aríñez-Soriano J., Cortés P., Maspoch D., Llagostera M. (2017). Microencapsulation with alginate/CaCO3: A strategy for improved phage therapy. Sci. Rep..

[24] Merabishvili M., Vervaet C., Pirnay J.-P., De Vos D., Verbeken G., Mast J., Chanishvili N., Vaneechoutte M. (2013). Stability of Staphylococcus aureus Phage ISP after Freeze-Drying (Lyophilization). PLoS ONE.

[25] Vimr E.R., Troy F.A. (1985). Regulation of Sialic Acid Metabolism in *Escherichia coli*: Role of N-acylneuraminate pyruvate-lyase. J. Bacteriol..

[26] Scholl D., Adhya S., Merril C. (2005). Escherichia *coli* K1’s Capsule Is a Barrier to Bacteriophage T7. Appl. Environ. Microbiol..

[27] Scholl D., Merril C. (2005). The genome of bacteriophage K1F, a T7-like phage that has acquired the ability to replicate on K1 strains of *Escherichia coli*. J. Bacteriol..

[28] Møller-Olsen C., Ho S.F.S., Shukla R.D., Feher T., Sagona A.P. (2018). Engineered K1F bacteriophages kill intracellular *Escherichia coli* K1 in human epithelial cells. Sci. Rep..

[29] Mahony D.E., Bell P.D., Easterbrook K.B. (1985). Two Bacteriophages of Clostridium difficile. J. Clin. Microbiol..

[30] Goh S., Chang B.J., Riley T.V. (2005). Effect of phage infection on toxin production by Clostridium difficile. J. Med. Microbiol..

[31] Vinner G.K., Malik D.J. (2018). High precision microfluidic microencapsulation of bacteriophages for enteric delivery. Res. Microbiol..

[32] Briers Y., Miroshnikov K., Chertkov O., Nekrasov A., Mesyanzhinov V., Volckaert G., Lavigne R. (2008). The structural peptidoglycan hydrolase gp181 of bacteriophage phiKZ. Biochem. Biophys. Res. Commun..

[33] Vandenheuvel D., Singh A., Vandersteegen K., Klumpp J., Lavigne R., Van Den Mooter G. (2013). Feasibility of spray drying bacteriophages into respirable powders to combat pulmonary bacterial infections. Eur. J. Pharm. Biopharm..

[34] Lee S.W., Belcher A.M. (2004). Virus-based fabrication of micro- and nanofibers using electrospinning. Nano Lett..

[35] Puapermpoonsiri U., Spencer J., Van Der Walle C.F. (2009). A freeze-dried formulation of bacteriophage encapsulated in biodegradable microspheres. Eur. J. Pharm. Biopharm..

[36] Leung S.S.Y., Parumasivam T., Gao F.G., Carrigy N.B., Vehring R., Finlay W.H., Morales S., Britton W.J., Kutter E., Chan H.-K. (2016). Production of Inhalation Phage Powders Using Spray Freeze Drying and Spray Drying Techniques for Treatment of Respiratory Infections. Pharm. Res..

[37] Knezevic P., Obreht D., Curcin S., Petrusic M., Aleksic V., Kostanjšek R., Petrović O. (2011). Phages of Pseudomonas aeruginosa: Response to environmental factors and in vitro ability to inhibit bacterial growth and biofilm formation. J. Appl. Microbiol..

[38] Tang Z., Huang X., Sabour P.M., Chambers J.R., Wang Q. (2015). Preparation and characterization of dry powder bacteriophage K for intestinal delivery through oral administration. LWT Food Sci. Technol..

[39] Ketola A.E., Leppänen M., Turpeinen T., Papponen P., Strand A., Sundberg A., Arstila K., Retulainen E. (2019). Cellulose nanofibrils prepared by gentle drying methods reveal the limits of helium ion microscopy imaging. RSC Adv..

[40] Smith W., Huggins M.B., Shaw K.M. (1987). The Control of Experimental *Escherichia coli* Diarrhoea in Calves by Means of Bacteriophages. J. Gen. Microbiol..

[41] Wills Q.F., Kerrigan C., Soothill J.S. (2005). Experimental Bacteriophage Protection against Staphylococcus aureus Abscesses in a Rabbit Model Experimental Bacteriophage Protection against Staphylococcus aureus Abscesses in a Rabbit Model. Antimicrob. Agents Chemother..

[42] Biswas B., Adhya S., Washart P., Paul B., Trostel A.N., Powell B., Carlton R., Merril C.R. (2002). Bacteriophage Therapy Rescues Mice Bacteremic from a Clinical Isolate of Vancomycin-Resistant Enterococcus faecium. Infect. Immun..

[43] Cerveny K.E., DePaola A., Duckworth D.H., Gulig P.A. (2002). Phage Therapy of Local and Systemic Disease Caused by Vibrio vulnificus in Iron-Dextran-Treated Mice. Infect. Immun..

[44] Ibekwe V.C., Fadda H.M., McConnell E.L., Khela M.K., Evans D.F., Basit A.W. (2008). Interplay between intestinal pH, transit time and feed status on the in vivo performance of pH responsive ileo-colonic release systems. Pharm. Res..

