# Supplementary material for: Microencapsulation of Enteric Bacteriophages in a pH-Responsive Solid Oral Dosage Formulation Using a Scalable Membrane Emulsification Process
Source: Pharmaceutics. 2019 Sep 14;11(9):475. doi: 10.3390/pharmaceutics11090475 (PMC6781335; doi:10.3390/pharmaceutics11090475)
Supplement: Supplementary file 1 [file pharmaceutics-11-00475-s001.pdf]

pharmaceutics

Supplementary Materials: Microencapsulation of Enteric Bacteriophages in a pH-Responsive Solid Oral Dosage Formulation Using a Scalable Membrane Emulsification Process

Gurinder K. Vinner, Kerry Richards, Miika Leppanen, Antonia P. Sagona and Danish J. Malik

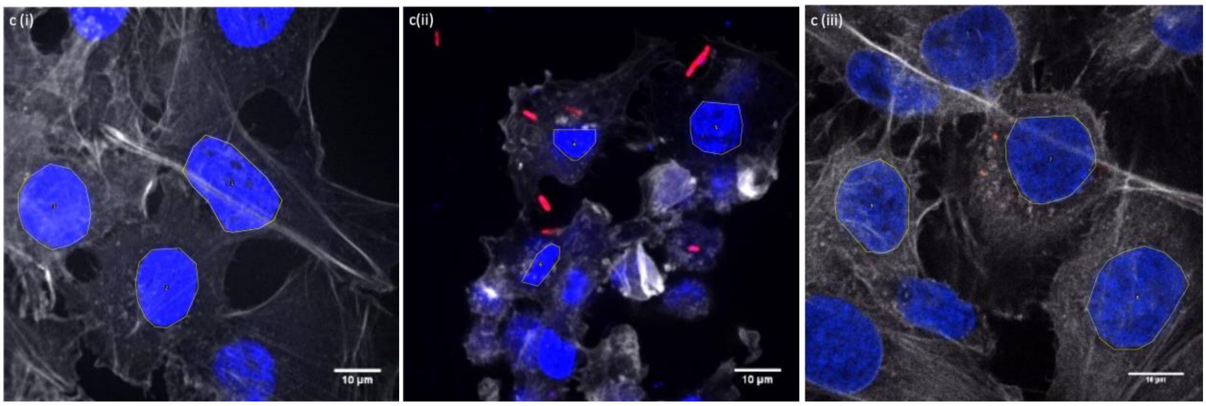

Figure S1. Measurement of cell nucleus perimeter using Fiji image processing software and calculation of nucleus area for each of the cells shown in Figure 8c.

Table S1. Summary of microencapsulated EKF1 storage results.

Storage Period/Weeks	Mean/PFU g^{-1}	Lower 95% CI	Upper 95% CI
0	[a] 1.8×10^{8}	8.9×10^{7}	2.8×10^{8}
1	[a] 1.1×10^{8}	8.4×10^{7}	1.3×10^{8}
2	[a] 1.1×10^{8}	8.4×10^{7}	1.3×10^{8}
3	[a] 8.3×10^{7}	5.0×10^{7}	1.2×10^{8}
4	[a] 8.8×10^{7}	6.0×10^{7}	1.1×10^{8}

Notes: CI = confidence interval; different superscripts in the same column indicate significantly different mean values at $p < 0.05$ (2-sample t-test) comparison with storage period 0 weeks.

Table S2. Summary of calculated areas of each cell nucleus. Cell no. refers to cells shown in Figure S1.

Cell no.	Nucleus Area
1	745
2	580
3	632
4	315
5	446
6	304
7	728
8	790
9	671
